# Supplementary material for: Cohesin activity accelerates the homology search
Source: bioRxiv. 2026 Jun 10:2026.06.08.730890. Preprint. [Version 1] doi: 10.64898/2026.06.08.730890 (PMC13278009; doi:10.64898/2026.06.08.730890)
Supplement: Supplementary file 1 [file NIHPP2026.06.08.730890v1-supplement-1.pdf]

bioRxiv preprint doi: https://doi.org/10.64898/2026.06.08.730890; this version posted June 10, 2026. The copyright holder for this preprint (which was not certified by peer review) is the author/funder, who has granted bioRxiv a license to display the preprint in perpetuity. It is made available under a CC-BY 4.0 International license.

SUPPLEMENTARY FIGURES

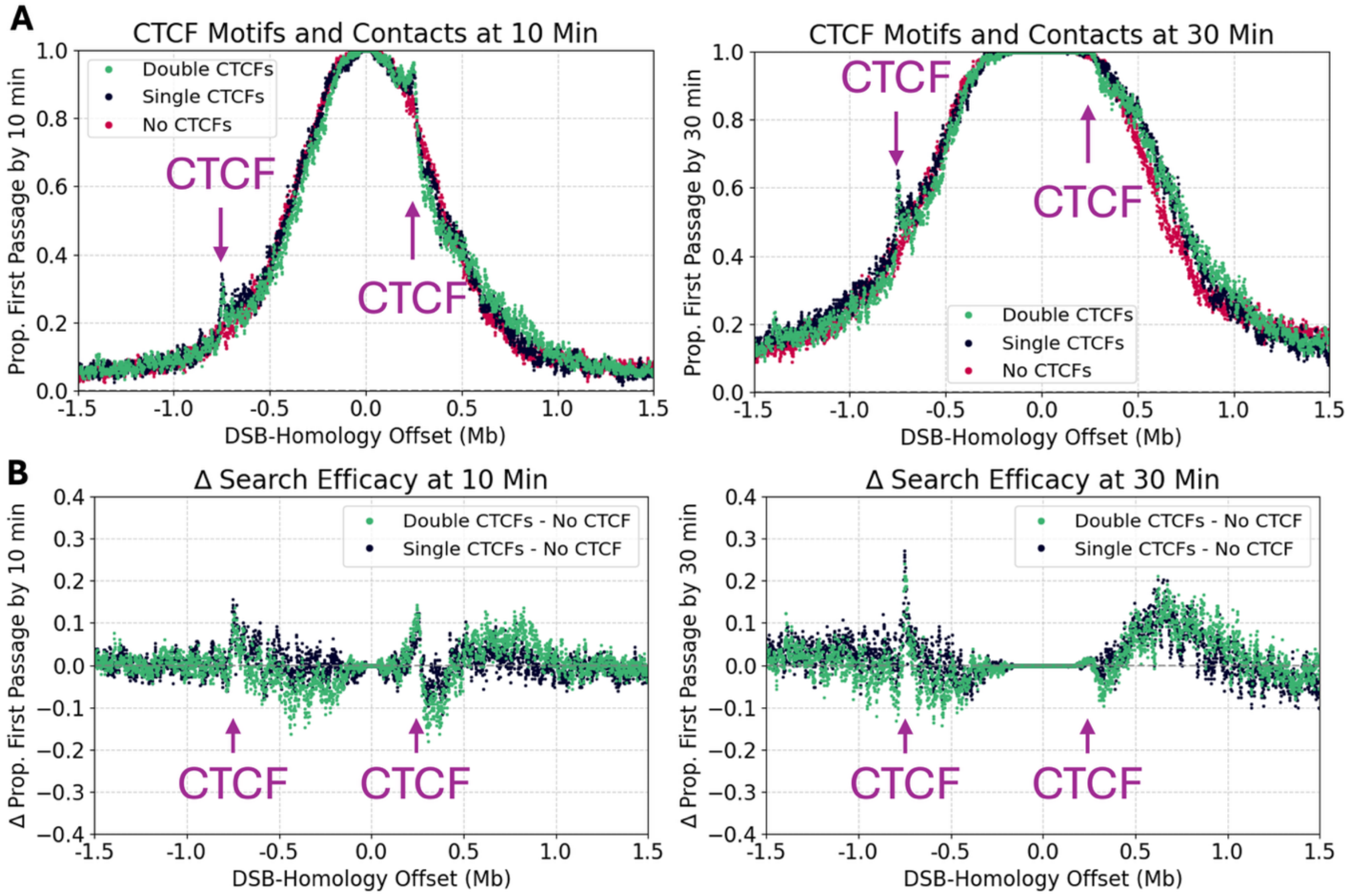

Supplementary Figure 1: CTCF and single-chromatid searches. *Single-chromatid simulations of DSB contact profiles on chromatids including boundary elements representing CTCF at -750 kb and +250 kb offsets from the break site. Positions of CTCFs are indicated by purple arrows. (A) effects of a single CTCF motif and of a double CTCF motif on first passage attainment profiles, in which two CTCFs are bound at a 10 kb separation from each other (-5 kb and +5kb of the original location). (B) presents the difference in first passage attainment between simulations including and not including CTCF motifs as a function of distance from the DSB site.*

bioRxiv preprint doi: https://doi.org/10.64898/2026.06.08.730890; this version posted June 10, 2026. The copyright holder for this preprint (which was not certified by peer review) is the author/funder, who has granted bioRxiv a license to display the preprint in perpetuity. It is made available under a CC-BY 4.0 International license.

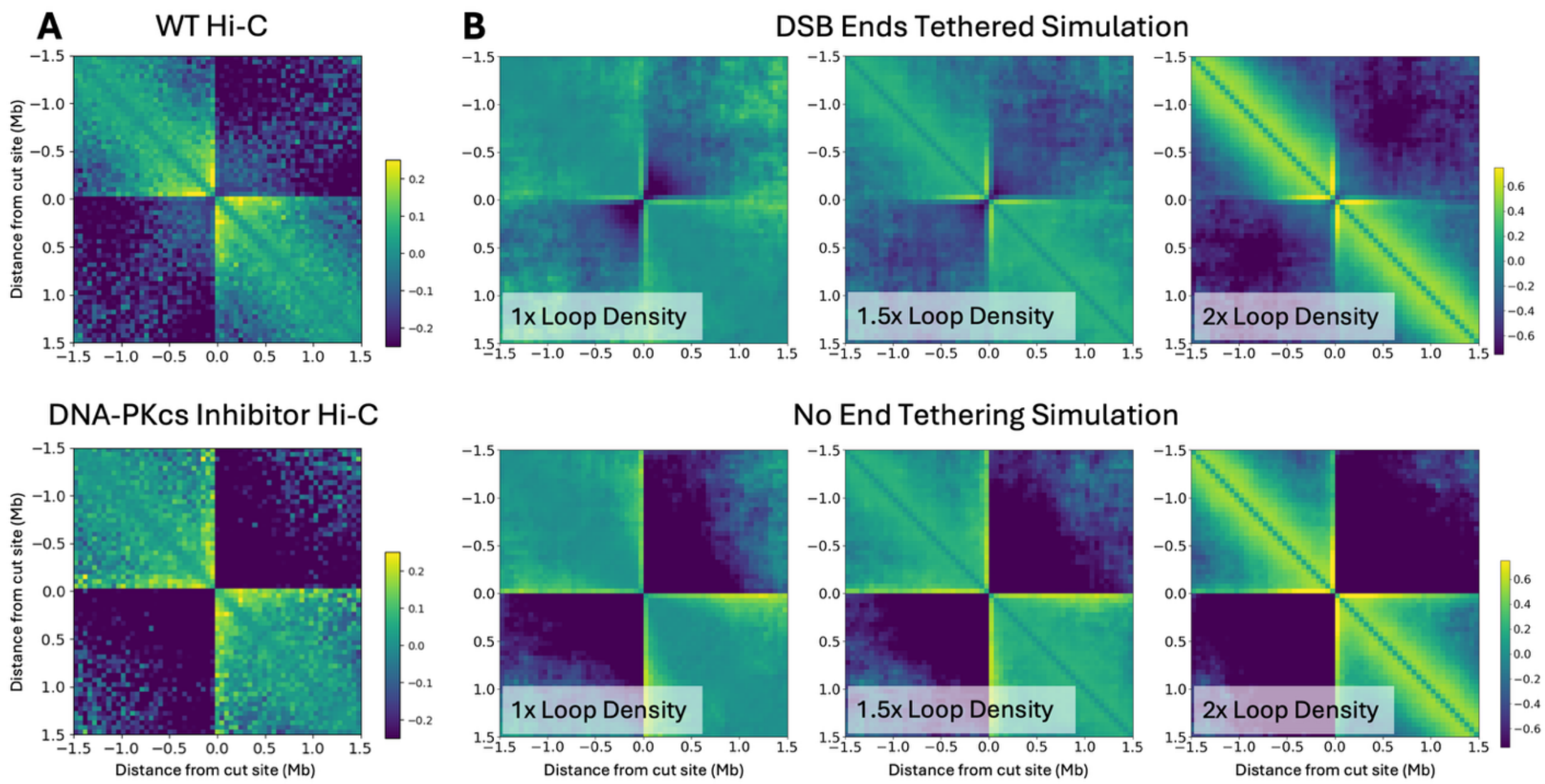

Supplementary Figure 2: Contact maps of single-chromatid searches. *(A) experimental Hi-C data from wild-type cells and cells with DNA-PKcs inhibited, previously reported in (8). PNA-PKcs is a protein associated with end tethering in NHEJ repair and is an early responder to DSBs. (B) simulation single-chromatid contact maps with and without DSB end tethering and with varying extents of post-break loop-extruding cohesin recruitment. End tethering was disabled by cleaving the bond between DSB ends, allowing them to diffuse freely in space. Loop-extruding cohesins were recruited immediately after the break to the specified total density. Simulations are across n=500 instances, distributed across 10 runs each containing a 150 Mb polymer segmented into 50 regions of 3 Mb each, with a DSB centered in each region.*

bioRxiv preprint doi: https://doi.org/10.64898/2026.06.08.730890; this version posted June 10, 2026. The copyright holder for this preprint (which was not certified by peer review) is the author/funder, who has granted bioRxiv a license to display the preprint in perpetuity. It is made available under a CC-BY 4.0 International license.

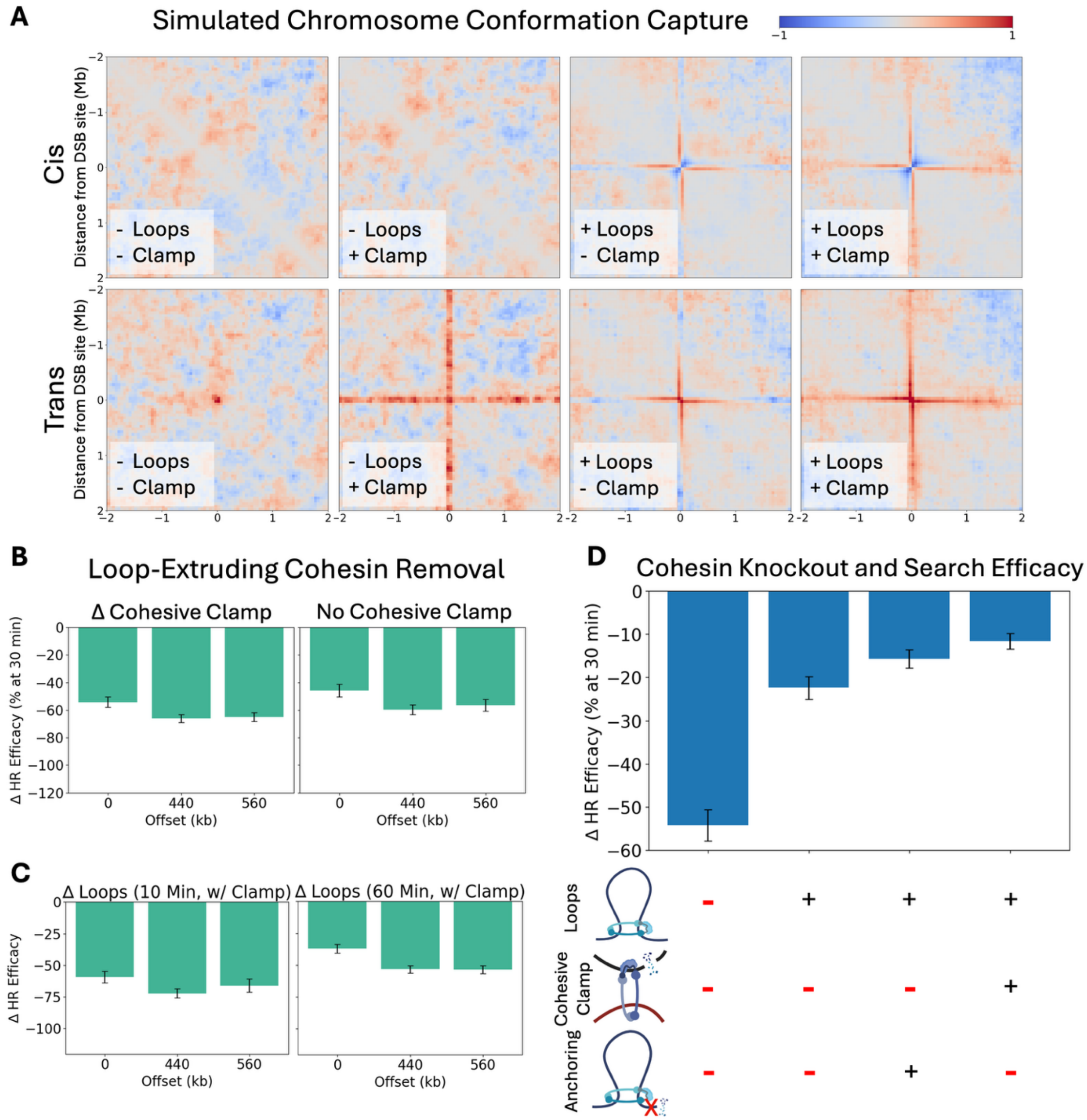

Supplementary Figure 3: Homology search contact maps and HR efficacy. *(A) contact maps from two-chromatid simulations with and without loop-extruding cohesins and cohesive clamps. (B) change in HR efficacy (first passage attainment) in simulations upon loop-extruding cohesin removal, with cohesive clamps either removed simultaneously with loop-extruding cohesins (left) or absent from both the control and the loop-extruding cohesin removal case (right). (C) change in HR efficacy at the 10- and 60-minute timepoints. (D) change in HR efficacy from removal of different combinations of loops, clamps, and anchoring from searches to the center of the sister chromatid (0 kb offset).*

bioRxiv preprint doi: https://doi.org/10.64898/2026.06.08.730890; this version posted June 10, 2026. The copyright holder for this preprint (which was not certified by peer review) is the author/funder, who has granted bioRxiv a license to display the preprint in perpetuity. It is made available under a CC-BY 4.0 International license.

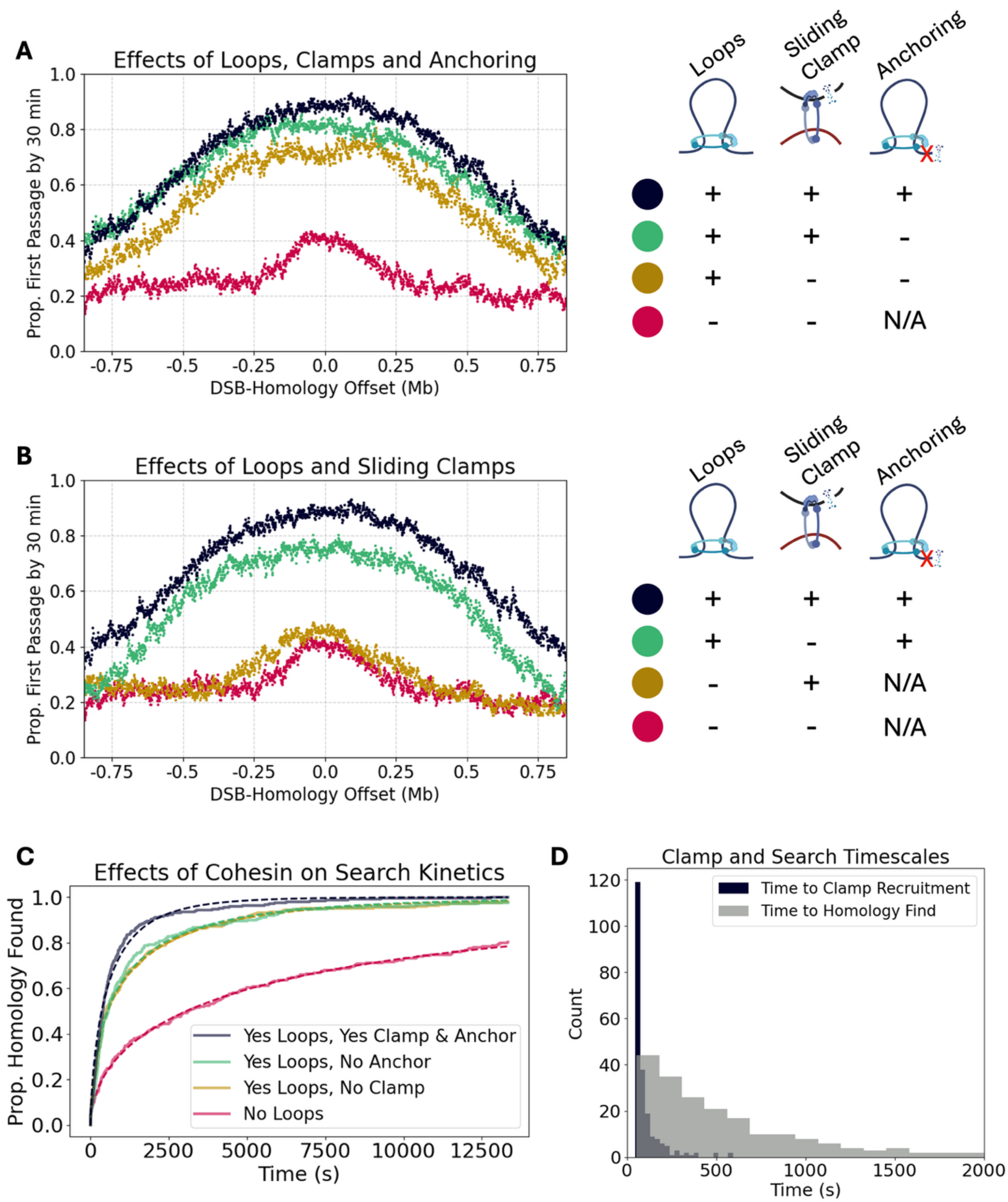

Supplementary Figure 4: Effects of various forms of cohesin on the homology search. *(A, B) homology search profile along the sister chromatid by position offset from the center of the sister chromatid, with various combinations of loops, cohesive clamps, and break-anchoring enabled. (C) effects of clamps and anchoring on search kinetics. (D) histogram of cohesive clamp binding time and homology passage time across n=218 simulation instances for a homology site centered*

bioRxiv preprint doi: https://doi.org/10.64898/2026.06.08.730890; this version posted June 10, 2026. The copyright holder for this preprint (which was not certified by peer review) is the author/funder, who has granted bioRxiv a license to display the preprint in perpetuity. It is made available under a CC-BY 4.0 International license.

in the sister chromatid. Simulation instances were drawn from an initial population of n=250, which were filtered to select simulations involving clamp binding prior to homology identification.

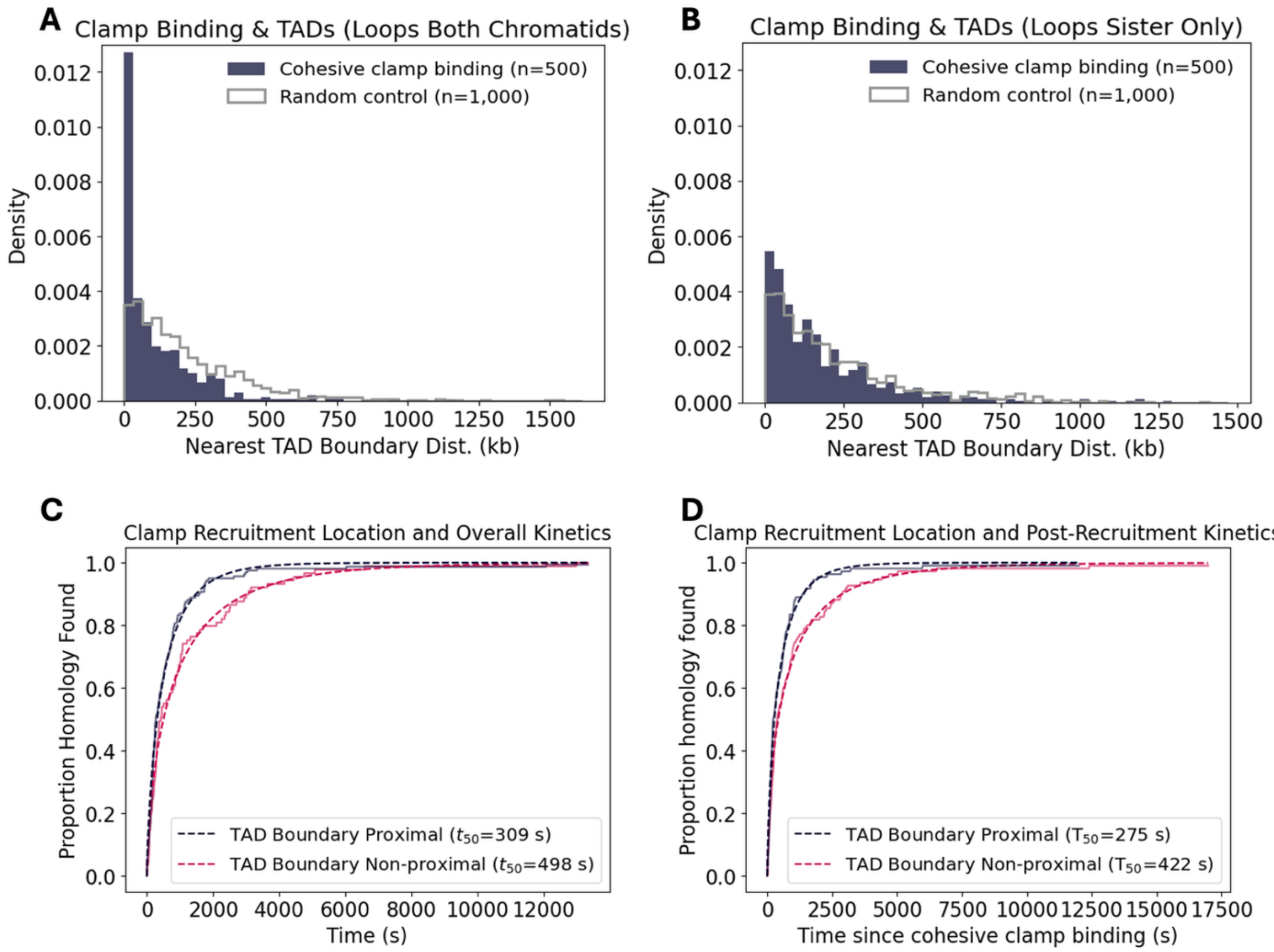

Supplementary Figure 5: Cohesive clamp recruitment and TAD boundary locations. *(C) a histogram of distances between template chromatid binding sites of DSB-recruited cohesive cohesins and their nearest TAD boundaries, with distances between TAD boundaries and randomly selected control positions in gray. (D) same histogram plotting method as (C) using data from a simulation with loop-extruding factors on the sister chromatid only. (C, D) search kinetics are graphed using a proximity threshold of 50 kb to classify DSB sites based on whether cohesive clamp recruitment events were proximal or non-proximal to TAD boundaries. (C) considers time between DSB induction and homology passage, while (D) considers time between clamp recruitment and homology passage. Homologies are assumed to be in the center of the sister chromatid. Simulation instances were drawn from an initial population of n=250, which were filtered to select simulations involving clamp binding prior to homology identification.*

bioRxiv preprint doi: https://doi.org/10.64898/2026.06.08.730890; this version posted June 10, 2026. The copyright holder for this preprint (which was not certified by peer review) is the author/funder, who has granted bioRxiv a license to display the preprint in perpetuity. It is made available under a CC-BY 4.0 International license.

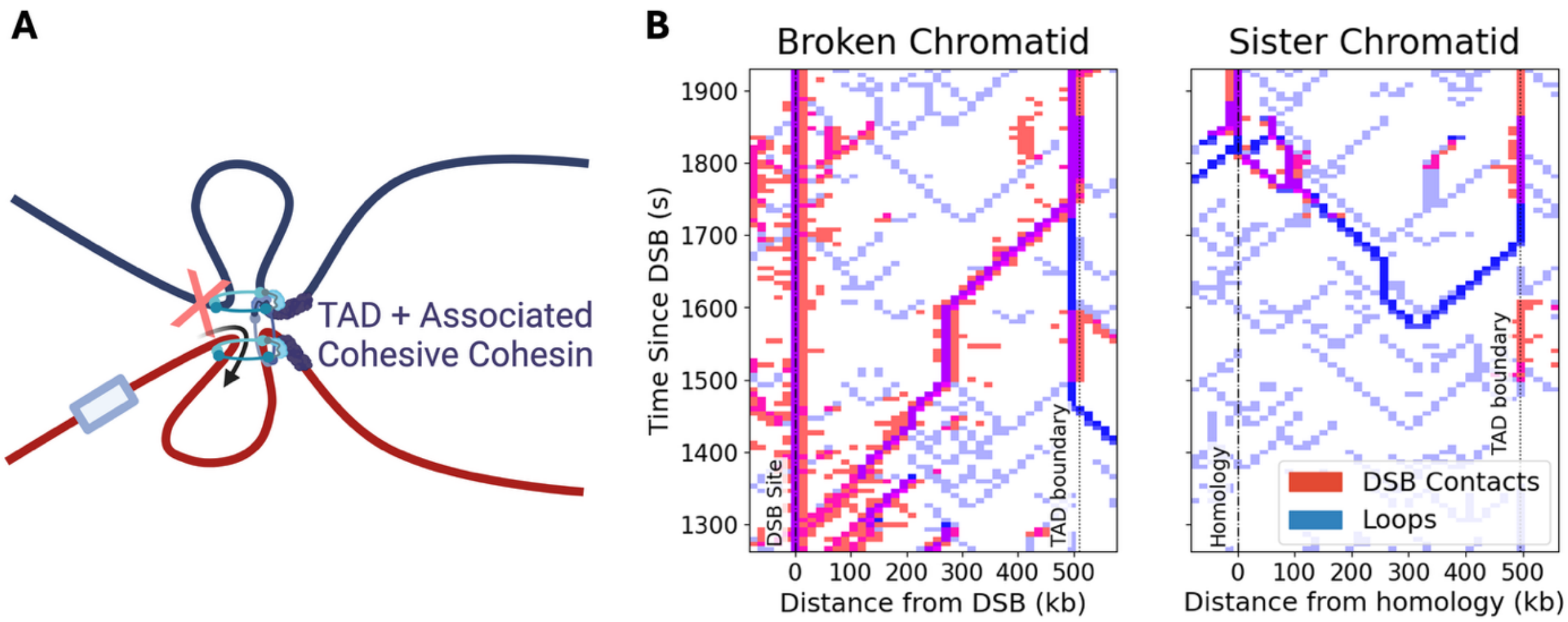

Supplementary Figure 6: Clamp-independent homology search mechanism. *(A) a model of a TAD-boundary-mediated sister chromatid search, where a broken chromatid loop-extruding cohesin loops the DSB site to a TAD boundary, enabling loop-extruding cohesins stalled at the TAD boundary on the sister chromatid to scan the DSB site along the chromatid. (B) a representative trace from a simulation with fixed TAD boundaries 500 kb from the break site (1 Mb TAD) and no cohesive clamp recruitment, showing such a mechanism.*

bioRxiv preprint doi: https://doi.org/10.64898/2026.06.08.730890; this version posted June 10, 2026. The copyright holder for this preprint (which was not certified by peer review) is the author/funder, who has granted bioRxiv a license to display the preprint in perpetuity. It is made available under a CC-BY 4.0 International license.

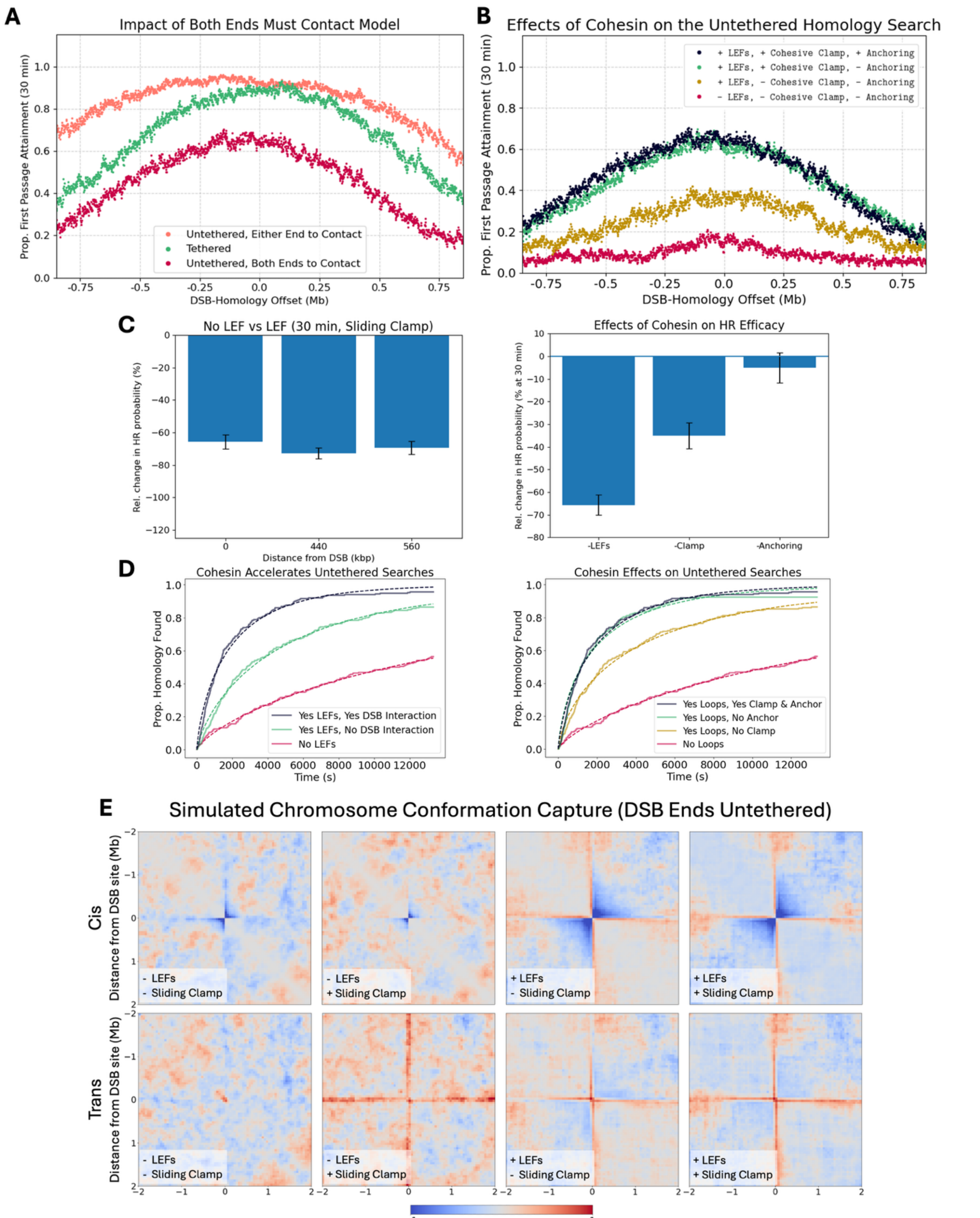

Supplementary Figure 7: The untethered search. *(A) impact of different models of loss of end tethering on the sister chromatid homology search profile. In the "both ends to contact" model, homology passage requires both DSB ends to have contacted the homology site, while "either end*

bioRxiv preprint doi: https://doi.org/10.64898/2026.06.08.730890; this version posted June 10, 2026. The copyright holder for this preprint (which was not certified by peer review) is the author/funder, who has granted bioRxiv a license to display the preprint in perpetuity. It is made available under a CC-BY 4.0 International license.

to contact" simply requires either end to have achieved passage. Where relevant, analysis in this figure requires both ends to have found the homology site to constitute a successful homology search. (B) effects of cohesin on the profile of the untethered homology search, showing a strong effect for loop-extruding cohesins and for cohesive clamps, but not for anchoring. (C) relative change in passage attainment for untethered searches upon loop-extruding cohesin removal. Various offsets between the sister chromatid center and the homology site are considered (left), as are the effects of cohesive clamp or anchoring removal on searches to the center of the sister chromatid (right), showing similar results to tethered searches except that break-anchoring no longer appears to improve search outcomes. (D) kinetics of untethered searches. (E) contact maps for untethered searches. n=250 for (A, B, C, D), n=500 for (E). "LEF" refers to loop-extruding factor, a term which describes loop-extruding cohesins. Loss of break-anchoring in untethered searches is here defined by unloading of loop-extruding cohesins at the break site ("falling off").
